# Transcription-wide impact by RESCUE-induced off-target single-nucleotide variants in mammalian cells

**DOI:** 10.1093/jmcb/mjad011

**Published:** 2023-02-23

**Authors:** Guo Li, Xiaoning Zhu, Yihan Wang, Hongru Ma, Yuzhe Wang, Hanyu Wu, Xiangyang Li, Yiling Wang, Jianen Gao, Xuexin Chen, Xingxu Huang, Yuan Yao, Xiaoxiang Hu

**Affiliations:** College of Chemical and Biological Engineering, Zhejiang University, Hangzhou 310027, China; ZJU-Hangzhou Global Scientific and Technological Innovation Center, Zhejiang University, Hangzhou 311200, China; State Key Laboratory of Agrobiotechnology, College of Biological Sciences, China Agricultural University, Beijing 100193, China; State Key Laboratory of Agrobiotechnology, College of Biological Sciences, China Agricultural University, Beijing 100193, China; National Research Institute for Family Planning, Beijing 100081, China; College of Chemical and Biological Engineering, Zhejiang University, Hangzhou 310027, China; ZJU-Hangzhou Global Scientific and Technological Innovation Center, Zhejiang University, Hangzhou 311200, China; State Key Laboratory of Agrobiotechnology, College of Biological Sciences, China Agricultural University, Beijing 100193, China; State Key Laboratory of Agrobiotechnology, College of Biological Sciences, China Agricultural University, Beijing 100193, China; School of Life Science and Technology, ShanghaiTech University, Shanghai 201210, China; ZJU-Hangzhou Global Scientific and Technological Innovation Center, Zhejiang University, Hangzhou 311200, China; National Research Institute for Family Planning, Beijing 100081, China; Institute of Insect Sciences, College of Agriculture and Biotechnology, Zhejiang University, Hangzhou 310058, China; Zhejiang Lab, Hangzhou 311121, China; College of Chemical and Biological Engineering, Zhejiang University, Hangzhou 310027, China; ZJU-Hangzhou Global Scientific and Technological Innovation Center, Zhejiang University, Hangzhou 311200, China; State Key Laboratory of Agrobiotechnology, College of Biological Sciences, China Agricultural University, Beijing 100193, China

**Keywords:** transcription-wide, off-target, SNV, RESCUE, RNA

## Abstract

RNA base editing is a promising tool in precise molecular therapy. Currently, there are two widely used RNA base editors, REPAIR and RESCUE. REPAIR only facilitates A-to-I conversions, while RESCUE performs both A-to-I and C-to-U conversions. Thus, RESCUE can generate twice the number of mutations compared to REPAIR. However, transcription-wide impact due to RESCUE-induced off-target single-nucleotide variants (SNVs) is not fully appreciated. Therefore, to determine the off-target effects of RESCUE-mediated editing, we employed transcription-wide sequencing on cells edited by RESCUE. The SNVs showed different off-target effects on mRNA, circRNA, lncRNA, and miRNA expression patterns and their interacting networks. Our results illustrate the transcription-wide impact of RESCUE-induced off-target SNVs and highlight the need for careful characterization of the off-target impact by this editing platform.

## Introduction

RNA base editing is a promising tool for cellular research and the treatment of genetic diseases (Savva et al., 2012). Two types of RNA base editors facilitate RNA base editing: REPAIR facilitates adenosine-to-inosine (A-to-I) conversions, while RESCUE performs both A-to-I and cytidine-to-uridine (C-to-U) conversions (Cox et al., 2017; Abudayyeh et al., 2019). The latter has been developed by fusing inactivated Cas13 (dCas13) with evolved ADAR2 (Abudayyeh et al., 2019). Thus, RESCUE-mediated RNA editing doubles the number of mutation types compared to REPAIR-mediated RNA editing. Since the editing to cytosine base generates substantial off-target single-nucleotide variants (SNVs) (Grünewald et al., 2019; Jin et al., 2019; Zuo et al., 2019), it is necessary to characterize the off-target impact of the RESCUE editing platform more carefully.

There are several types of RNA molecules in a cell. mRNA is a linear, single-stranded molecule and typically mediates the translation of DNA into proteins. Circular RNA (circRNA) forms a covalently closed continuous loop and is typically represented in the eukaryotic transcriptome (Awasthi et al., 2018). MicroRNA (miRNA) is the small endogenous RNA that regulates post-transcriptional gene expression (Lu and Rothenberg, 2018). Long non-coding RNA (lncRNA) is often <200 bp in length and rarely codes for a protein (Quinn and Chang, 2016). These RNAs form an interacting network and play different roles in development and disease. For instance, circRNAs regulate transcription and mRNA turnover (Vo et al., 2018; Chen et al., 2019; Liu et al., 2019). Individual circRNA molecules act as miRNA sponges and have a regulatory role in transcription and translation (Panda, 2018). circRNAs are unusually stable forms of circularized exons (Capel et al., 1993; Hansen et al., 2013). During RNA editing, A-to-I base conversion antagonizes circRNA production by restricting the formation of a covalently closed continuous loop (Memczak et al., 2013). Thus, it is of utmost importance to assess the transcription-wide impact of RESCUE-induced off-target SNVs in mammalian cells.

High-resolution sequencing tools such as Digenome-seq (Kim et al., 2015), whole-genome sequencing (Grünewald et al., 2019; Zuo et al., 2019), and RNA sequencing (RNA-seq) (Jin et al., 2019) have been successfully employed in off-target investigation. Here, we employed RNA-seq to decipher the off-target effects on mRNA, circRNA, lncRNA, and miRNA expression patterns and their interacting networks. Our results reveal that RESCUE induces lots of A-to-I and C-to-U off-target SNVs affecting mRNA, circRNA, and lncRNA expression and their interacting networks, which illustrates that safety assessment and editing tool improvement are necessary for research and future clinical applications.

## Results

### ADAR2 in RESCUE induces off-target SNVs

**Figure 1:**
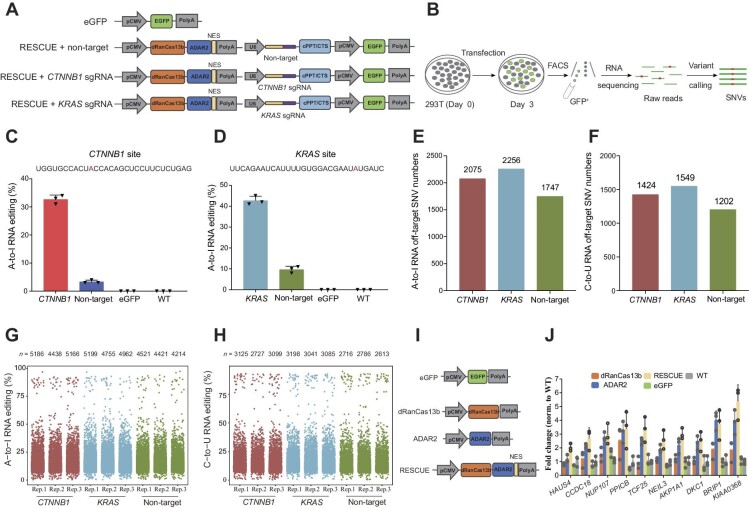
Transcriptome-wide off-target A-to-I and C-to-U RNA editing induced by RESCUE in 293T cells. (**A**) Schematic illustration of the mammalian expression constructs. EGFP, the control group; RESCUE + non-target, the non-target RNA editing group; RESCUE + *CTNNB1* sgRNA, the *CTNNB1* RNA editing group; RESCUE + *KRAS* sgRNA, the *KRAS* RNA editing group. (**B**) Transcriptome-sequencing experimental design. (**C** and **D**) The A-to-I RNA editing efficiency of *CTNNB1* mRNA (**C**) or *KRAS* mRNA (**D**) mediated by RESCUE RNA editor. (**E** and **F**) The statistics of total A-to-I (**E**) or C-to-U (**F**) off-target SNVs appeared in *CTNNB1, KRAS*, and non-target editing groups. (**G** and **H**) Manhattan plots of A-to-I (**G**) and C-to-U (**H**) off-target SNVs in *CTNNB1, KRAS*, and non-target editing groups. *n*, total number of modified adenines (**G**) or cytosines (**H**). (**I**) Schematic illustration of the mammalian expression constructs ADAR2, dRanCas13b, RESCUE, and eGFP. (**J**) RT–qPCR assays quantifying changes in circRNAs.

RESCUE is a highly efficient RNA editing tool that facilitates both A-to-I and C-to-U conversions. It was developed by directly evolving ADAR2 deaminase domain (ADAR2dd) into a cytidine deaminase and subsequently fusing it to an inactive Cas13b ortholog from *Riemerella anatipestifer* (dRanCas13b). To assess RESCUE-induced off-target SNVs, we engineered *CTNNB1* and *KRAS*, two previously reported test oncogenes for RESCUE editing (Abudayyeh et al., 2019), as target genes. To evaluate the editing effect, we established four experimental groups and transfected the plasmids into 293T cells as: RESCUE with *CTNNB1* sgRNA, RESCUE with *KRAS* sgRNA, RESCUE with non-target sgRNA, and eGFP, respectively (Figure 1A). At 48 h post-transfection, 1 × 10^6^ GFP-positive cells were collected and analyzed by fluorescence-activated cell sorting (FACS) (Figure 1B).

Total RNA from these cells, as well as wild-type (WT) 293T cells, was extracted for high-resolution transcriptome sequencing with 20× coverage (Figure 1B). The average A-to-I RNA editing efficiencies from three replicates were 32.7% for *CTNNB1* (Figure 1C) and 42.7% for *KRAS* (Figure 1D). For the RNA off-target SNVs, there were 2075 A-to-I and 1424 C-to-U conversions in *CTNNB1*, 2256 A-to-I and 1549 C-to-U conversions in *KRAS*, and 1747 A-to-I and 1202 C-to-U conversions in the non-target editing group (Figure 1E and F). A total of 1385 A-to-I and 1000 C-to-U off-target SNVs appeared in both *CTNNB1* and *KRAS* editing groups, respectively (Supplementary Figure S1). Furthermore, A-to-I and C-to-U SNVs distributed uniformly in chromosomal regions, as indicated by the distribution patterns (Figure 1G and H; Supplementary Figure S2).

**Figure 2:**
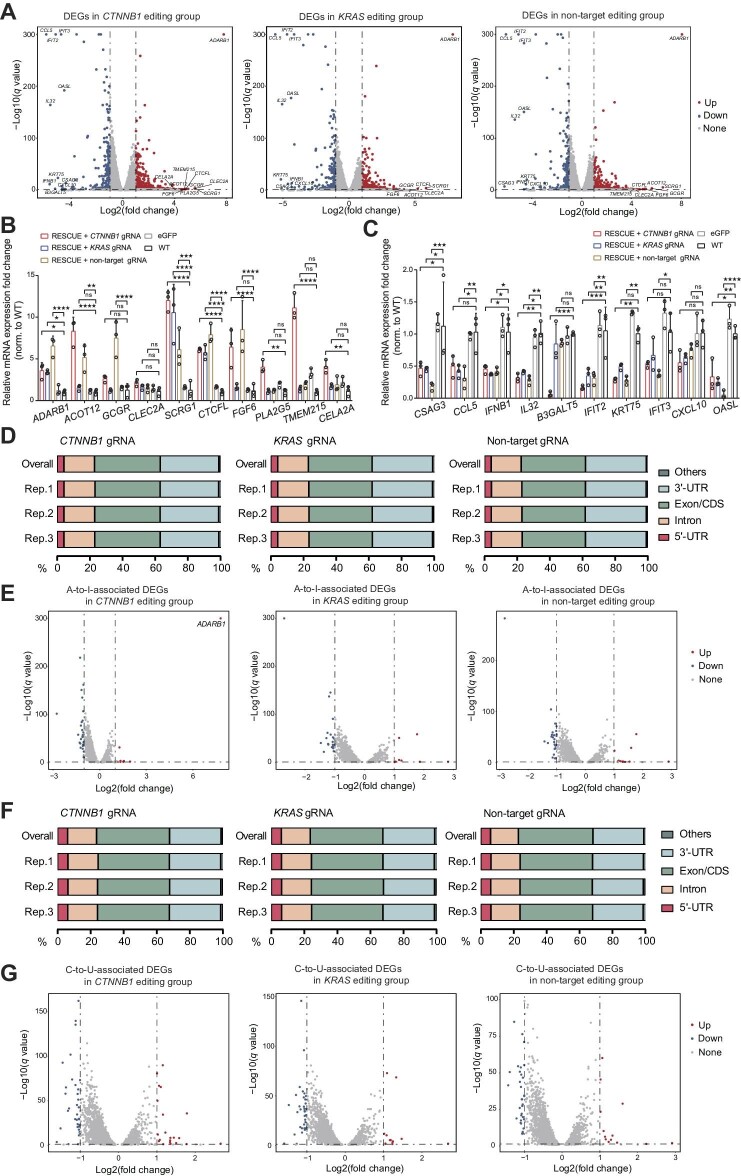
Identification of DEGs with A-to-I or C-to-U off-target SNVs. (**A**) Volcano plots of the total DEGs in *CTNNB1, KRAS*, and non-target editing groups. Log2(fold change) > 1, *q* value < 0.05, and ‘*q* value = 0’ was represented by ‘*q* value = 1E−300’. (**B** and **C**) Relative expression fold change of the top 10 up-regulated (**B**) and down-regulated (**C**) mRNAs among *CTNNB1, KRAS*, and non-target editing groups. (**D** and **F**) The distributions of mRNA A-to-I (**D**) and C-to-U (**F**) off-target SNVs in *CTNNB1, KRAS*, and non-target editing groups. Others represent the ‘Up 10 kb’ and ‘Down 10 kb’ mRNA regions. (**E** and **G**) Volcano plots of the DEGs with A-to-I (**E**) and C-to-U (**G**) off-target SNVs in *CTNNB1, KRAS*, and non-target editing groups. *P* < 0.05, FDR < 0.5, and mutation frequencies of the three eGFP samples were all 0.

### RESCUE-induced off-target SNVs affect mRNA expression

By analyzing the RNA-seq data, we found that RESCUE-mediated RNA editing resulted in differential gene expression patterns.

In *CTNNB1, KRAS*, and non-target editing groups, we observed 878 (609 up-regulated and 269 down-regulated), 655 (407 up-regulated and 258 down-regulated), and 790 (520 up-regulated and 270 down-regulated) differentially expressed genes (DEGs), respectively (Figure 2A). We further examined the relative expression of the top 10 up-regulated (Figure 2B) and down-regulated (Figure 2C) DEGs, respectively, by reverse transcription–quantitative polymerase chain reaction (RT–qPCR) (Supplementary Table S4). The results showed that *ADARB1, ACOT12, GCGR, CLEC2A, SCRG1, CTCFL, FGF6, PLA2G5, TMEM215*, and *CELA2A* in the *CTNNB1* editing group, *ADARB1, SCRG1*, and *CTCFL* in the *KRAS* editing group, and *ADARB1, SCRG1, ACOT12, GCGR, FGF6*, and *CTCFL* in the non-target editing group were all up-regulated with up to 12-fold change in expression (Figure 2B), while *CSAG3, CCL5, IFNB1, IL32, B3GALT5, IFIT2, KRT75, IFIT3, CXCL10*, and *OASL* in the *CTNNB1* editing group, *CSAG3, CCL5, IFNB1, IL32, IFIT2, KRT75, IFIT3, CXCL10*, and *OASL* in the *KRAS* editing group, and *CSAG3, CCL5, IFNB1, IL32, IFIT2, KRT75, IFIT3, CXCL10*, and *OASL* in the non-target editing group were all down-regulated with up to 6-fold change in expression (Figure 2C), compared with that in WT and GFP expression groups.

To test whether RNA off-target SNVs could also regulate RNA expression, we examined RESCUE-induced A-to-I- and C-to-U-associated differentially expressed RNAs (Supplementary Tables S5 and S6). As shown in Supplementary Figure S4A and C, the numbers of differentially expressed RNAs were 1898, 1648, and 1893 (from three triplicates) associated with A-to-I and 2278, 2000, and 2275 associated with C-to-U in the *CTNNB1* editing group, 1944, 1797, and 1831 associated with A-to-I and 2332, 2209, and 2243 associated with C-to-U in the *KRAS* editing group, and 1644, 1501, and 1893 associated with A-to-I and 1970, 2044, and 1909 associated with C-to-U in the non-target editing group, respectively. There was no difference in either A-to-I or C-to-U RNA editing efficiency between differentially expressed RNAs associated and unassociated with SNVs (Supplementary Figure S3). Moreover, among *CTNNB1, KRAS*, and non-target editing groups, there were 741 A-to-I-associated differentially expressed RNAs, including 730 DEGs, seven differentially expressed circRNAs (DECs), and four differentially expressed lncRNAs (DELs) (Supplementary Figure S4A–D) and 1950 C-to-U-associated differentially expressed RNAs, including 1934 DEGs, 6 DECs, and 10 DELs (Supplementary Figure S4E–G).

Furthermore, we found that A-to-I and C-to-U off-target SNVs were mainly located in exons, untranslated regions (3′-UTRs), and introns of mRNAs (Figure 2D and F) and in exons and 3′-UTRs of DEGs (Supplementary Figure S5A–D). The exon-located A-to-I off-target SNVs were made up of 68.43% silent mutations and 31.57% missense mutations in the *CTNNB1* editing group, 67.30% silent mutations and 32.70% missense mutations in the *KRAS* editing group, and 67.89% silent mutations and 32.11% missense mutations in the non-target editing group, respectively (Supplementary Figure S5E). The exon-located C-to-U off-target SNVs were made up of 50.83% missense mutations, 45.54% silent mutations, and 3.63% nonsense mutations in the *CTNNB1* editing group, 50.62% missense mutations, 45.81% silent mutations, and 3.57% nonsense mutations in the *KRAS* editing group, and 50.82% missense mutations, 45.86% silent mutations, and 3.32% nonsense mutations in the non-target editing group (Supplementary Figure S5F).

These off-target SNV-associated DEGs showed different expression patterns. There were a total of 37 (10 up-regulated and 27 down-regulated), 28 (eight up-regulated and 20 down-regulated), and 36 (11 up-expressed and 25 down-expressed) A-to-I-associated DEGs in *CTNNB1, KRAS*, and non-target editing groups (Figure 2E). Interestingly, *ADARB1* was significantly up-regulated in all three groups but only associated with A-to-I off-target SNVs in the *CTNNB1* editing group (Figure 2A and E). There were a total of 55 (21 up-regulated and 34 down-regulated), 48 (12 up-regulated and 36 down-regulated), and 45 (13 up-regulated and 32 down-regulated) C-to-U off-target SNV-associated DEGs in *CTNNB1, KRAS*, and non-target editing groups, respectively (Figure 2G). Then, we randomly selected seven A-to-I- and nine C-to-U-associated DEGs in the *CTNNB1* editing group and detected their expression by qPCR. Results showed that all seven A-to-I-associated whereas eight C-to-U-associated mRNAs were significantly up- or down-regulated compared with the WT control (Supplementary Figure S5G and H). Function and pathway characterization by Kyoto Encyclopaedia of Genes and Genomes (KEGG) Orthology database revealed that these DEGs were mainly related to ‘cytokine–cytokine receptor interaction’ (Supplementary Figure S6).

These results demonstrate that RESCUE-mediated RNA base editing generates numerous DEGs, some of which are associated with RESCUE-induced A-to-I and C-to-U off-target SNVs.

### RESCUE-induced off-target SNVs modulate circRNA expression

A previous study illustrated that depletion of both *ADAR* and *DHX9* enhanced double-stranded RNA accumulation defects, leading to increased circRNA production (Aktaş et al., 2017). Additionally, the CRISPR endoribonuclease Csy4 acts as an activator of circularization (Borchardt et al., 2017). Since RESCUE is comprised of ADAR2 deaminase and CRISPR-associated endoribonuclease (dRanCas13b), we inferred that RESCUE might impact circular RNA production. To validate this assumption, we transfected expression plasmids, dRanCas13b, ADAR2, RESCUE, and eGFP (Figure 1I), respectively, into 293T cells and checked the abundance of circRNAs (previously reported circRNA candidates as indicators; Xiong et al., 2018) by RT–qPCR at 48 h post-transfection. We found that RESCUE contributed to circRNA formation, as evidenced by the robustly increased circRNA levels in ADAR2- and RESCUE-transfected cells (Figure 1J). Then, we analyzed different expression patterns of DECs in *CTNNB1, KRAS*, and non-target editing groups.

**Figure 3:**
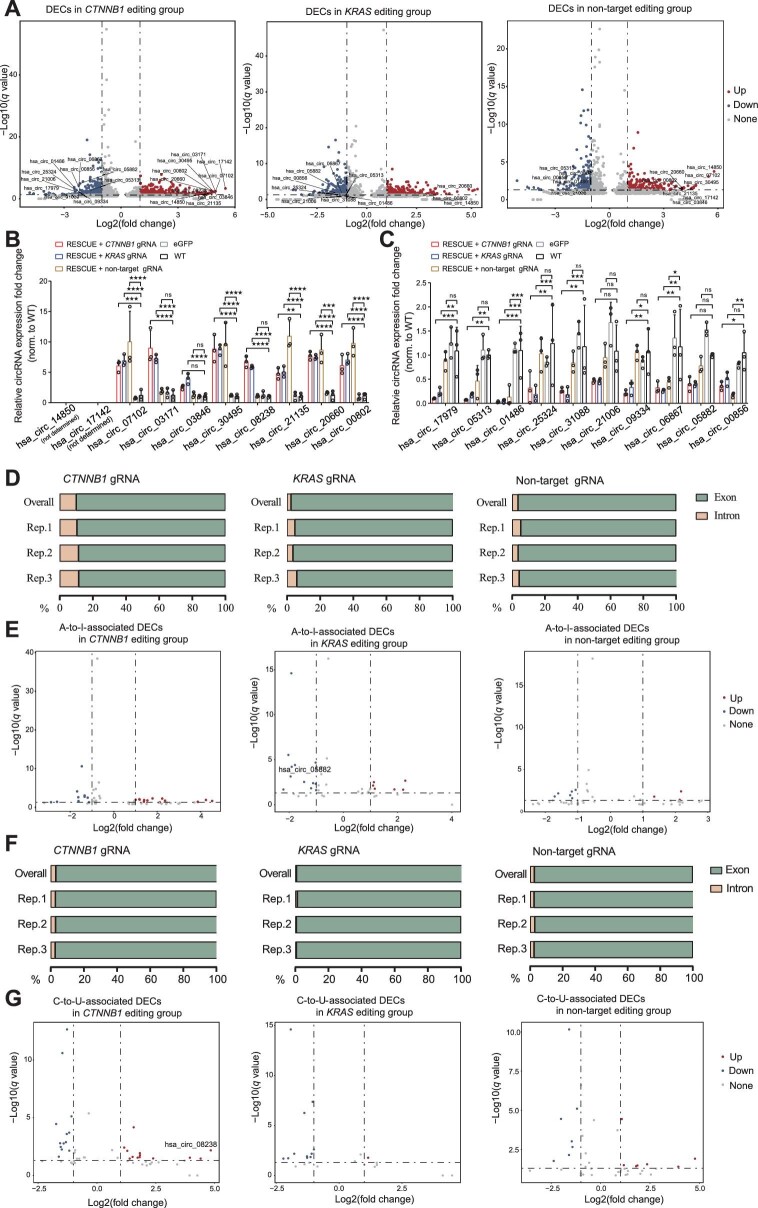
Identification of DECs with A-to-I or C-to-U off-target SNVs. (**A**) Volcano plots of the total DECs in *CTNNB1, KRAS*, and non-target editing groups. Log2(fold change) > 1, *q* value < 0.05. (**B** and **C**) Relative expression fold change of the top 10 up-regulated (**B**) and down-regulated (**C**) circRNAs among *CTNNB1, KRAS*, and non-target editing groups. (**D** and **F**) The distributions of circRNA A-to-I (**D**) and C-to-U (**F**) off-target SNVs in *CTNNB1, KRAS*, and non-target editing groups. (**E** and **G**) Volcano plots of the DECs with A-to-I (**E**) and C-to-U (**G**) off-target SNVs in *CTNNB1, KRAS*, and non-target editing groups. *P* < 0.05, FDR < 0.5, and mutation frequencies of the three eGFP samples were all 0.

A total of 652 (396 up-regulated and 256 down-regulated). 492 (260 up-regulated and 232 down-regulated), and 371 (197 up-regulated and 174 down-regulated) DECs were observed in *CTNNB1, KRAS*, and non-target editing groups (Figure 3A). We further analyzed the relative expression of the top 10 up-regulated (Figure 3B) and down-regulated (Figure 3C) DECs, respectively, by RT–qPCR (Supplementary Table S4). The results showed that hsa_circ_07102, hsa_circ_03171, hsa_circ_03846, hsa_circ_30495, hsa_circ_08238, hsa_circ_21135, hsa_circ_20660, and hsa_circ_00802 in both *CTNNB1* and *KRAS* editing groups and hsa_circ_07102, hsa_circ_30495, hsa_circ_21135, hsa_circ_20660, and hsa_circ_00802 in the non-target editing group were all up-regulated with up to 8-fold change in expression (Figure 3B), while hsa_circ_17979, hsa_circ_05313, hsa_circ_01486, hsa_circ_25324, hsa_circ_31088, hsa_circ_21006, hsa_circ_09334, hsa_circ_06867, hsa_circ_05882, and hsa_circ_00856 in both *CTNNB1* and *KRAS* editing groups and hsa_circ_01486, hsa_circ_05313, hsa_circ_06867, and hsa_circ_00856 in the non-target editing group were all down-regulated by up to 7-fold (Figure 3C), compared with that in WT and GFP expression groups.

To test whether RNA off-target SNVs could also regulate DECs, we examined A-to-I- and C-to-U-associated DECs induced by RESCUE. We found that circRNA A-to-I and C-to-U off-target SNVs were mainly located in exons (Figure 3D and F; Supplementary Figure S7A and B). Further characterization revealed 23 (13 up-regulated and 10 down-regulated), 19 (six up-regulated and 13 down-regulated), and 10 (four up-regulated and six down-regulated) A-to-I off-target SNV-associated DECs in *CTNNB1, KRAS*, and non-target editing groups, respectively (Figure 3E). On the other hand, the numbers of DECs with C-to-U RNA off-target SNVs were 26 (13 up-regulated and 13 down-regulated), 12 (one up-regulated and 11 down-regulated), and 14 (seven up-regulated and seven down-regulated) in *CTNNB1, KRAS*, and non-target editing groups, respectively (Figure 3G). Then, we selected seven A-to-I-, and nine C-to-U-associated DECs in the *CTNNB1* editing group and detected their expression by qPCR. Results showed that all seven A-to-I-associated whereas six C-to-U-associated circRNAs were significantly up- or down-regulated compared with the WT control (Supplementary Figure S7C and D).

These results demonstrate that RESCUE-mediated RNA base editing generates several DECs, some of which are associated with RESCUE-induced A-to-I and C-to-U off-target SNVs.

### RESCUE-induced off-target SNVs modulate lncRNA expression

Given the impact of widespread induction of RNA off-target SNVs, we investigated how DELs, which play a significant role in gene function, were associated with off-target SNVs (Xiong et al., 2018).

**Figure 4:**
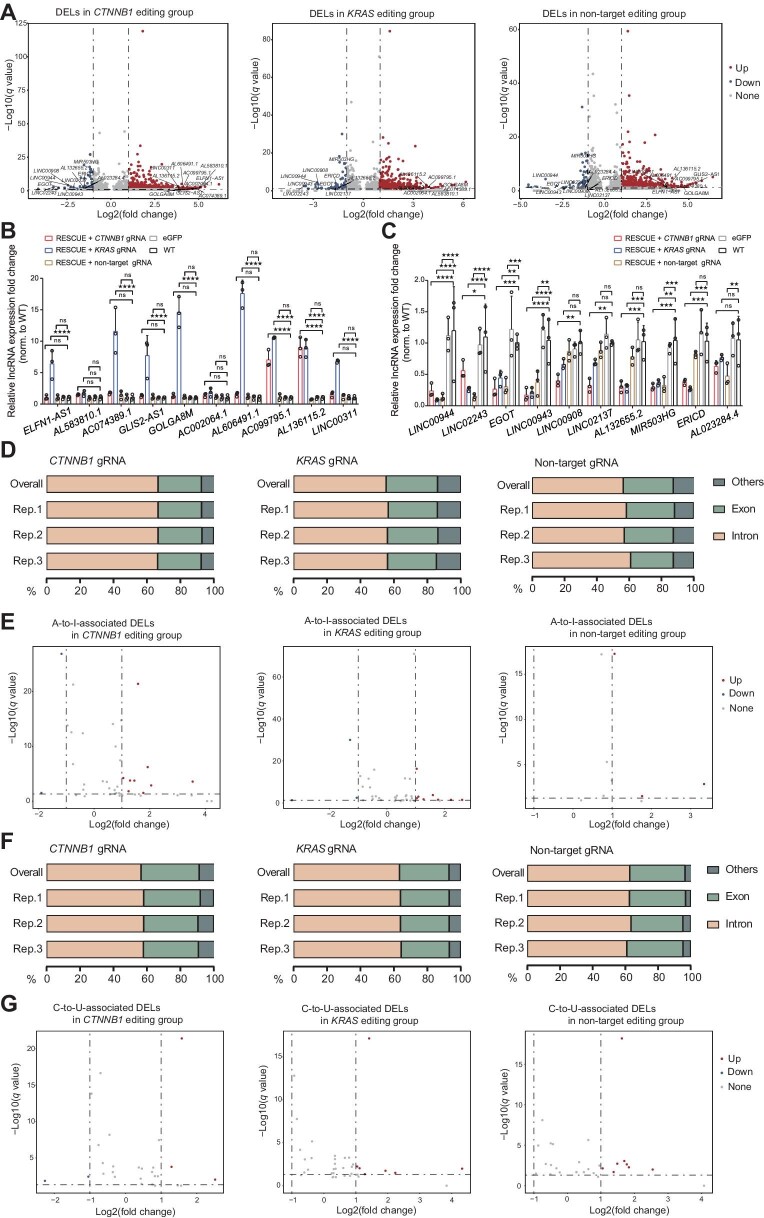
Identification of DELs with A-to-I or C-to-U off-target SNVs. (**A**) Volcano plots of the total DELs in *CTNNB1, KRAS*, and non-target editing groups. Log2(fold change) > 1, *q* value < 0.05. (**B** and **C**) Relative expression fold change of the top 10 up-regulated (**B**) and down-regulated (**C**) lncRNAs among *CTNNB1, KRAS*, and non-target editing groups. (**D** and **F**) The distributions of lncRNA A-to-I (**D**) and C-to-U (**F**) off-target SNVs in *CTNNB1, KRAS*, and non-target editing groups. (**E** and **G**) Volcano plots of the DELs with A-to-I (**E**) and C-to-U (**G**) off-target SNVs in *CTNNB1, KRAS*, and non-target editing groups. *P*  < 0.05, FDR < 0.5, and mutation frequencies of the three eGFP samples were all 0.

Analysis of the RNA-seq data revealed a total of 556 (494 up-regulated and 62 down-regulated), 472 (402 up-regulated and 70 down-regulated), and 501 (444 up-regulated and 57 down-regulated) DELs in *CTNNB1, KRAS*, and non-target editing groups, respectively (Figure 4A). We further analyzed the relative expression of the top 10 up-regulated (Figure 4B) and down-regulated (Figure 4C) DELs, respectively, by qPCR (Supplementary Table S4). The results showed that *ELFN1-AS1, AL583810.1, AC074389.1, GLIS2-AS1, GOLGA8M, AC002064.1, AL606491.1, AC099795.1, AL136115.2*, and *LINC00311* in the *CTNNB1* editing group, *AL136115.2* and *AC099795.1* in the *KRAS* editing group, and *ELFN1-AS1, AC074389.1, GLIS2-AS1, GOLGA8M, AL606491.1, AC099795.1, AL136115.2*, and *LINC00311* in the non-target editing group were all up-regulated by up to 10-fold (Figure 4B), while *LINC00944, LINC02243, EGOT, LINC00943, LINC00908, LINC02137, AL132655.2, MIR503HG, ERICD*, and *AL023284.4* in the *CTNNB1* editing group, *LINC00944, LINC02243, EGOT, LINC00943, LINC00908, LINC02137, AL132655.2, MIR503HG*, and *ERICD* in the *KRAS* editing group, and *LINC00944, LINC02243, EGOT, LINC00943, MIR503HG*, and *AL023284.4* in the non-target editing group were all down-regulated by up to 10-fold (Figure 4C).

To test whether RNA off-target SNVs also regulate DELs, we examined A-to-I- and C-to-U-associated DELs induced by RESCUE. We found that lncRNA A-to-I and C-to-U off-target SNVs were mainly located in introns (Figure 4D and F; Supplementary Figure S8A and B). There were 11 (nine up-regulated and two down-regulated), 12 (nine up-regulated and three down-regulated), and three up-regulated DELs associated with A-to-I off-target SNVs in *CTNNB1, KRAS*, and non-target editing groups, respectively (Figure 4E). Meanwhile, there were six (three up-regulated and three down-regulated), six (all up-regulated), and eight (all up-regulated) DELs associated with C-to-U off-target SNVs in *CTNNB1, KRAS*, and non-target editing groups, respectively (Figure 4G). Then, we selected 10 A-to-I- and six C-to-U-associated DELs in the *CTNNB1* editing group and detected their expression by qPCR. Results showed that nine A-to-I-associated whereas three C-to-U-associated lncRNAs were significantly up- or down-regulated compared with the WT control (Supplementary Figure S8C and D).

These results demonstrate that RESCUE-mediated RNA base editing generates numerous DELs, some of which are associated with RESCUE-induced A-to-I and C-to-U off-target SNVs. Therefore, RESCUE-induced off-target SNVs modulate the expression of mRNA, circRNA, and lncRNA.

### RNA off-target SNVs influence RNA interacting networks

CircRNAs function as miRNA sponges and play a regulatory role in transcription and translation. miRNAs are small endogenous RNAs that regulate gene expression post-transcriptionally (Panda, 2018). In this study, we found 16, 24, and 14 differentially expressed miRNAs (DEMs) in *CTNNB1, KRAS*, and non-target editing groups, respectively (Supplementary Figure S9). However, we did not identify any A-to-I or C-to-U off-target SNVs located in any of these DEMs.

**Figure 5:**
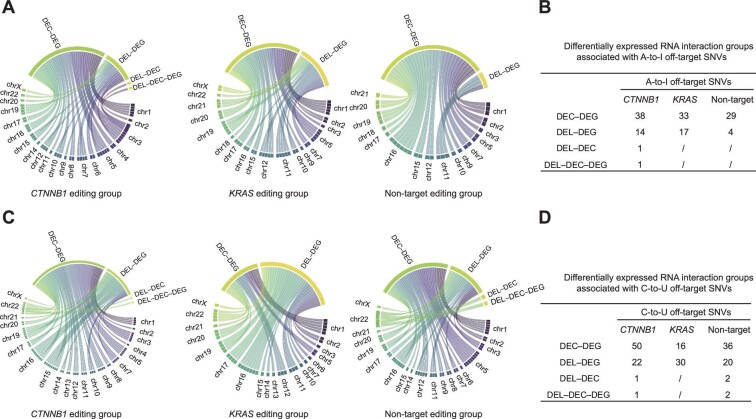
Differentially expressed RNA interactions associated with A-to-I and C-to-U off-target SNVs. (**A** and **B**) The chord diagrams and total numbers of differentially expressed RNA interactions associated with A-to-I off-target SNVs in *CTNNB1, KRAS*, and non-target editing groups. (**C** and **D**) The chord diagrams and total numbers of differentially expressed RNA interactions associated with C-to-U off-target SNVs in *CTNNB1, KRAS*, and non-target editing groups.

We analyzed RNA interacting networks to determine the interactions among different types of RNA. For samples associated with A-to-I off-target SNVs, we found 38 DEC–DEG, 14 DEL–DEG, one DEL–DEC, and one DEL–DEC–DEG interactions in the *CTNNB1* editing group, 33 DEC–DEG and 17 DEL–DEG interactions in the *KRAS* editing group, and 29 DEC–DEG and 4 DEL–DEG interactions in the non-target editing group (Figure 5A and B; Supplementary Table S7). For samples associated with C-to-U off-target SNVs, we found 50 DEC–DEG, 22 DEL–DEG, one DEL–DEC, and one DEL–DEC–DEG interactions in the *CTNNB1* editing group, 16 DEC–DEG and 30 DEL–DEG interactions in the *KRAS* editing group, and 36 DEC–DEG, 20 DEL–DEG, 2 DEL–DEC, and 2 DEL–DEC–DEG interactions in the non-target editing group (Figure 5C and D; Supplementary Table S8). These comparative accumulations of interactions illustrate that A-to-I and C-to-U off-target SNVs change the expression patterns and interacting networks among mRNA, circRNA, miRNA, and lncRNA species.

## Discussion

Our research clearly illustrates the impact of off-target SNVs on mRNA, circRNA, miRNA, and lncRNA interacting networks. The RNA editing tool RESCUE induces A-to-I and C-to-U off-target SNVs. These changes and their impact need to be characterized more carefully for wider biomedical applications. The ADAR enzymes of the RESCUE system cause A-to-I conversions that destabilize intron base-pairing interactions, impair pre-mRNA looping, and diminish exon circularization (Bolha et al., 2017). C-to-U conversions may facilitate circRNA formation by promoting structure looping or exon circularization. RESCUE-induced circRNA formation may regulate gene expression by mediating transcription, mRNA turnover, and miRNA translation (Memczak et al., 2013).

miRNAs repress mRNA expression and protein production by destabilizing target mRNAs and binding to their 3′-UTRs (Boucas et al., 2012). Therefore, the circRNA–miRNA–mRNA networks play a crucial role in biological processes, especially in post-transcriptional regulation. Previous studies suggest that the circRNA–miRNA–mRNA axis is involved in human disease signaling pathways by regulating pathogenicity-related gene expression. For instance, circRNA and miRNA participate in the pathological development of cardiovascular diseases (Li et al., 2019). CircRNA–miRNA–mRNA networks may be a novel regulatory mechanism for effective coronary heart disease (Lin et al., 2020). In addition, circRNA profiles may serve as candidates for the diagnosis of non-alcoholic steatohepatitis (NASH) and may provide new insights into NASH and hepatocellular carcinoma pathophysiology (Jin et al., 2016). Thus, we propose that fluctuations in the circRNA–miRNA–mRNA networks dysregulate the transcriptome and post-transcriptional gene expression. Our research provides evidence of the altered circRNA–miRNA–mRNA networks by RESCUE-induced off-target SNVs, which might significantly affect the biological development and disease process.

Extensive research is required to improve the safety of RNA editing tools and their potential clinical applications. Previous studies have highlighted improvements in the specificities by RESCUE. For example, the DD-Cas9 system, which fuses an FKBP12-derived destabilizing domain to Cas9, enables the conditional Cas9 expression and temporal control of gene editing in an FKBP12 synthetic ligand (Senturk et al., 2017). Such a system might be a suitable candidate for improving the specificity of RESCUE by reducing the effective time of ADAR2 deaminase. A new RNA base editor, REPAIRx, which has the deaminase domain of ADAR2 inserted into the middle of CasRx (RfxCas13d), leading to a decrease in the off-target A-to-I RNA editing via the REPAIR RNA editor system, has been reported (Liu et al., 2020). Such strategies could be taken advantage of to reduce RNA off-target SNVs of the RESCUE system involving ADAR2 and dRanCas13b. These two studies highlight new approaches to improve RESCUE into an advanced tool.

In conclusion, our study demonstrates that RESCUE-induced off-target SNVs influence RNA expression and their interacting networks. Our findings illustrate that safety assessment and editing tool improvement are necessary for research and future clinical applications.

## Materials and methods

### Design and cloning of mammalian constructs for RNA editing

We purchased several plasmids for RNA editing from Addgene (https://www.addgene.org/). pC0078 RESCUE (#130661) (Abudayyeh et al., 2019) contains dRanCas13b–ADAR2dd (RESCUE) with a C-terminal mapkNES fusion. Guide RNAs containing 3′ direct repeats and compatible with RanCas13b were cloned into the *Bbs*I site of pC0041-RanCas13b crRNA backbone (#103852) (Cox et al., 2017).

### gRNA design and construction

We selected two sites, *CTNNB1*, TGGTGCCACTACCACAGCTC CTTCTCTGAG (A/11), and *KRAS*, TTCAGAATCATTTTGTGGAC GAATATGATC (A/25), to design the sgRNA for RNA A-to-I editing where the base A would be replaced by G (Supplementary Table S2). We designed a 30-bp sgRNA sequence for the two target sites and cloned it as a C-terminal GFP fusion into the *Bbs*I site of the RanCas13b crRNA backbone.

### Mammalian cell culture and transfection

293T cells were maintained in Dulbecco's modified Eagle medium (DMEM, 4.5 g/L glucose) supplemented with 10% fetal bovine serum (GE Life Sciences) at 37°C with 5% CO_2_. Upon reaching 70%–80% confluency, the cells were dissociated using TrypLE Express (Life Technologies) and passaged at a ratio of 1:2. On the day before transfection, 293T cells were trypsinized, counted, and plated at 1.5 × 10^6^ cells per well in 2.5 ml of complete growth medium. The cells were ∼80% confluent on the day of transfection, and the growth medium was removed and replaced with 2.5 ml fresh complete growth medium. For transfection, 3.0 µg DNA and 9.0 µl EZ Trans Reagent (Shanghai Life Bio-Tech Co., Ltd, #C4058L1092) were diluted in 120 µl DMEM, respectively, mixed gently, and incubated for 20 min at room temperature to form DNA–EZ Trans Reagent complexes before directly added to the cells and mixed gently by rocking the plate back and forth. After 6 h, the complexes were removed, and 2.5 ml of complete growth medium was added to the cells for another 48-h incubation at 37°C in a 5% CO_2_ incubator.

### Preparation of RNA-seq samples

GFP-positive cells were collected by FACS at 48 h after transfection. Approximately 1 × 10^6^ cells per sample were collected in a 15-ml tube containing 500 µl DMEM (4.5 g/L glucose) supplemented with 10% fetal bovine serum. Total RNA was then extracted using TRIzol reagent to obtain 10–15 µg RNA per sample. RNA degradation and contamination were monitored on 1% agarose gels. RNA purity was checked using a NanoPhotometer^®^ spectrophotometer (IMPLEN). RNA integrity was assessed using an RNA Nano 6000 Assay Kit of the Bioanalyzer 2100 system (Agilent Technologies).

### Transcriptome sequencing (novogene)

For RNA library preparations, 1 µg total RNA per sample was used as input material. Following the manufacturer's recommendations, sequencing libraries were generated using NEBNext^®^ Ultra^™^ RNA Library Prep Kit for Illumina^®^ (NEB #7530), and index codes were added to attribute sequences to each sample. Briefly, mRNA was purified from total RNA using poly-T oligo-attached magnetic beads. Using divalent cations under elevated temperature, fragmentation was carried out in NEBNext First Strand Synthesis Reaction Buffer (5×). First-strand cDNA was synthesized using random hexamer primer and M-MuLV reverse transcriptase (RNase H−). Second-strand cDNA synthesis was subsequently performed using DNA polymerase I and RNase H. Remaining overhangs were converted into blunt ends via exonuclease/polymerase activities. After adenylation of 3′ ends of DNA fragments, NEBNext Adaptor with hairpin loop structure was ligated to prepare for hybridization. In order to select cDNA fragments of preferentially 250–300 bp in length, the library fragments were purified with the AMPure XP system (Beckman Coulter). Then, 3 µl USER Enzyme (NEB) was used with size-selected, adaptor-ligated cDNA at 37°C for 15 min, followed by 5 min at 95°C before PCR was performed with Phusion High-Fidelity DNA polymerase, universal PCR primers, and index (X) primer. At last, PCR products were purified (AMPure XP system), and library quality was assessed on the Agilent Bioanalyzer 2100 system.

According to the manufacturer's instructions, the clustering of the index-coded samples was performed on a cBot Cluster Generation System using TruSeq PE Cluster Kit v3-cBot-HS (Illumia). After cluster generation, the library preparations were sequenced on an Illumina Novaseq platform, and 150-bp paired-end reads were generated.

### miRNA sequencing (novogene)

For small RNA library preparations, 3 µg total RNA per sample was used as input material. Sequencing libraries were generated using NEBNext^®^ Multiplex Small RNA Library Prep Set for Illumina^®^ (NEB #E7330) following manufacturer's recommendations, and index codes were added to attribute sequences to each sample. Briefly, NEB 3′ SR Adaptor was directly ligated to 3′ ends of miRNA, siRNA, and piRNA. Then, the SR RT primer hybridized to the excess of 3′ SR Adaptor (that remained free after the 3′ ligation reaction), followed by 5′ SR Adapter ligated to 5′ ends of miRNA, siRNA, and piRNA. First-strand cDNA was synthesized using M-MuLV reverse transcriptase (RNase H−). PCR amplification was performed using LongAmp Taq 2× Master Mix, SR Primer for Illumina, and index (X) primer. PCR products were purified on an 8% polyacrylamide gel (100 V, 80 min). DNA fragments corresponding to 140–160 bp (the length of small non-coding RNA plus the 3′ and 5′ adaptors) were recovered and dissolved in 8 µl elution buffer. At last, library quality was assessed on the Agilent Bioanalyzer 2100 system using DNA High Sensitivity Chips.

According to the manufacturer's instructions, the clustering of the index-coded samples was performed on a cBot Cluster Generation System using TruSeq SR Cluster Kit v3-cBot-HS (Illumia). After cluster generation, the library preparations were sequenced on an Illumina Hiseq 2500/2000 platform, and 50-bp single-end reads were generated.

### circRNA sequencing (novogene)

For RNA library preparations, 5 µg total RNA per sample was used as input material. Firstly, ribosomal RNA was removed by Epicentre Ribo-zero^™^ rRNA Removal Kit, and rRNA-free residue was cleaned up by ethanol precipitation. Subsequently, the linear RNA was digested with RNase R (Epicentre), 3 U for 1 µg RNA. Following manufacturer's recommendations, the sequencing libraries were generated by NEBNext^®^ Ultra^™^ Directional RNA Library Prep Kit for Illumina^®^ (NEB #7420). Briefly, fragmentation was carried out using divalent cations under elevated temperature in NEBNext First Strand Synthesis Reaction Buffer (5×). First-strand cDNA was synthesized using random hexamer primer and M-MuLV reverse transcriptase (RNase H−). Second-strand cDNA synthesis was subsequently performed using DNA polymerase I and RNase H. In the reaction buffer, dNTPs with dTTP were replaced by those with dUTP. The remaining overhangs were converted into blunt ends via exonuclease/polymerase activities. After adenylation of 3′ ends of DNA fragments, NEBNext Adaptor with hairpin loop structure was ligated to prepare for hybridization. In order to select cDNA fragments of preferentially 150–200 bp in length, the library fragments were purified with the AMPure XP system. Then, 3 µl USER Enzyme was used with size-selected, adaptor-ligated cDNA at 37°C for 15 min, followed by 5 min at 95°C before PCR was performed with Phusion High-Fidelity DNA polymerase, universal PCR primers, and index (X) primer. At last, products were purified (AMPure XP system), and library quality was assessed on the Agilent Bioanalyzer 2100 system.

According to the manufacturer's instructions, the clustering of the index-coded samples was performed on a cBot Cluster Generation System using TruSeq PE Cluster Kit v3-cBot-HS. After cluster generation, the libraries were sequenced on an Illumina Hiseq 4000 platform, and 150-bp paired-end reads were generated.

### SNV genotyping

SNVs were called following the best practices from GATK (version 4.1.9) for the RNA-seq data. Clean reads were used to perform local realignment, coordinate sorting, base quality score recalibration, and indel realignment. We performed SNV discovery and used multi-sample variant calling to distinguish between a homozygous reference genotype and a missing genotype among the analyzed samples. SNVs were annotated using the transcript set from the Homo_sapiens_Ensemble_94 version.

### Whole-transcriptome analysis

Quality control and filtering of raw data with default parameters were performed using fastp (v0.20.1). The clean reads were mapped to hg38 (human) using hisat2 (v2.0.4) with the options ‘hisat2—dta—new-summary -t -p 6 -x index -1 fq1 -2 fq2 -S sample.sam’. Then, SAMtools (v1.9) was used to convert the SAM files to BAM format for sorting to quantify the expression of different types of RNA. For mRNA and lncRNA, HTSeq (v0.12.3) was used, and gene expression count was normalized as fragments per kilobase of exon model per million mapped fragments (FPKM). For circRNA, the reads mapped to the genome were filtered out, and then circRNA information was downloaded from the circRNADb database (http://reprod.njmu.edu.cn/circrnadb/doc/circRNA_dataset.zip) to build a circRNA database to align the remaining reads. According to the alignment, the number of reads in each circRNA was counted, and the expression of circRNA was quantified by reads per million reads. The mapped reads were finally extracted and aligned to human miRNAs in the miRBase database (https://www.mirbase.org/). The miRNA expression was normalized by tag per million reads (TPM).

### Differential expression analysis

Differentially expressed mRNA, lncRNA, circRNA, and miRNA sets between pairwise comparisons were identified using the DESeq2 (v1.30.1) package in R (v4.0.3). Threshold for significantly different expression was set at as: log2(fold change) > 1 and *q* value < 0.05. Hierarchical clustering analysis and KEGG pathway analysis were performed using R (v4.0.3). Scatter, heatmap, and histogram plots were plotted using R (v4.0.3).

### Correlation analysis between DEGs, DECs, or DELs and off-target SNVs

The results of the 20× standard were selected for downstream analysis, with the number and list of off-target SNVs in each experimental group. Follow-up studies were conducted with each gene as a unit, and 10000 off-target SNVs inside, upstream, and downstream of the gene were extracted. The correlation analysis was carried out according to mutation frequencies of these off-target SNVs and different gene expression types. The correlation test was significant (*P* < 0.05, FDR < 0.5; the mutation frequencies of the three eGFP samples were all 0).

### RT–qPCR

To verify the expression of different types of RNA, we detected the relative expression of mRNA, circRNA, and lncRNA by RT–qPCR using ChamQ Universal SYBR qPCR Master Mix (Vazyme #Q711). Total RNA of the GFP-positive cells was extracted using the TRIzol reagent. cDNA was synthesized by using oligo d(T) primers and used as the template for SYBR Green-based real-time PCR. The reaction was performed at 95°C for 2 min, followed by 40 cycles of 95°C for 15 sec and 61°C for 1 min using an ABI 7300 detection system. The standard curve method was used for quantification, and the cDNA of detected mRNA was 10-fold serially diluted to generate the standard curve. mRNA quantities of the samples were determined by linear extrapolation of the Ct values plotted against the standard curve. All the assays were repeated for at least three times, and each experiment was performed in triplicate. One-way or two-way ANOVA with multiple comparison corrections was used to assess the statistical significance of transcript changes using Prism 7. The qPCR primers of mRNA, circRNA, and lncRNA are illustrated in Supplementary Tables S1 and S3.

## Supplementary Material

mjad011_Supplemental_Files

## Data Availability

RNA editing efficiency statistics: EditR (https://moriaritylab.shinyapps.io/editr_v10/).

## Notes

SNVs annotated: Homo_sapiens_Ensemble_94 version.

## Notes

SNVs calling: GATK (version 4.1).

## Notes

Raw data filtering: Fastp (v0.20.1).

## Notes

Gene expression quantification: HTSeq (v0.12.3).

## Notes

Gene expression count normalization: FPKM.

## Notes

miRNA expression normalization: TPM.

## Notes

Differential mRNA, lncRNA, circRNA, and miRNA analysis: DESeq2 (v1.30.1).

## Notes

Hierarchical clustering analysis and KEGG pathway analysis: R (v4.0.3).

## Notes

The RNA-seq data will be deposited at NCBI Bioproject (PRJNA808167).

## Notes

**Author contributions:** G.L., Y.Y., X. Huang, and X. Hu conceived and designed the project. G.L. performed most experiments with the assistance of Yihan Wang, H.W., and X.L. X.Z. and Yuzhe Wang analyzed most of the RNA-seq data with the assistance of H.M. and Yiling Wang. X. Hu, Y.Y., X. Huang, and J.G. supervised the project. X.C. provided assistance in experimental facilities. X.Z. and Yihan Wang should be regarded as joint first authors.

## References

[bib1] Abudayyeh O.O. , Gootenberg J.S., Franklin B. et al. (2019). A cytosine deaminase for programmable single-base RNA editing. Science 365, 382–386. 31296651 10.1126/science.aax7063 PMC6956565

[bib2] Aktaş T. , Avşar Ilık İ., Maticzka D. et al. (2017). DHX9 suppresses RNA processing defects originating from the Alu invasion of the human genome. Nature 544, 115–119. 28355180 10.1038/nature21715

[bib3] Awasthi R. , Singh A.K., Mishra G. et al. (2018). An overview of circular RNAs. Adv. Exp. Med. Biol. 1087, 3–14. 30259353 10.1007/978-981-13-1426-1_1

[bib4] Bolha L. , Ravnik-Glavač M., Glavač D. (2017). Circular RNAs: biogenesis, function, and a role as possible cancer biomarkers. Int. J. Genomics 6218353. 29349062 10.1155/2017/6218353 PMC5733622

[bib5] Borchardt E.K. , Meganck R.M., Vincent H.A. et al. (2017) Inducing circular RNA formation using the CRISPR endoribonuclease Csy4. RNA 23, 619–627. 28223408 10.1261/rna.056838.116 PMC5393173

[bib6] Boucas J. , Riabinska A., Jokic M. et al. (2012). Posttranscriptional regulation of gene expression-adding another layer of complexity to the DNA damage response. Front. Genet. 3, 159. 22936947 10.3389/fgene.2012.00159 PMC3427493

[bib7] Capel B. , Swain A., Nicolis S. et al. (1993). Circular transcripts of the testis-determining gene Sry in adult mouse testis. Cell 73, 1019–1030. 7684656 10.1016/0092-8674(93)90279-y

[bib8] Chen S. , Huang V., Xu X. et al. (2019). Widespread and functional RNA circularization in localized prostate cancer. Cell 176, 831–843.e22. 30735634 10.1016/j.cell.2019.01.025

[bib9] Cox D.B.T. , Gootenberg J.S., Abudayyeh O.O. et al. (2017). RNA editing with CRISPR–Cas13. Science 358, 1019–1027. 29070703 10.1126/science.aaq0180 PMC5793859

[bib10] Grünewald J. , Zhou R., Garcia S.P. et al. (2019). Transcriptome-wide off-target RNA editing induced by CRISPR-guided DNA base editors. Nature 569, 433–437. 30995674 10.1038/s41586-019-1161-z PMC6657343

[bib11] Hansen T.B. , Jensen T.I., Clausen B.H. et al. (2013). Natural RNA circles function as efficient microRNA sponges. Nature 495, 384–388. 23446346 10.1038/nature11993

[bib12] Jin S. , Zong Y., Gao Q. et al. (2019). Cytosine, but not adenine, base editors induce genome-wide off-target mutations in rice. Science 364, 292–295. 30819931 10.1126/science.aaw7166

[bib13] Jin X. , Feng C.Y., Xiang Z. et al. (2016). CircRNA expression pattern and circRNA–miRNA–mRNA network in the pathogenesis of nonalcoholic steatohepatitis. Oncotarget 7, 66455–66467. 27677588 10.18632/oncotarget.12186 PMC5341813

[bib14] Kim D. , Bae S., Park J. et al. (2015). Digenome-seq: genome-wide profiling of CRISPR–Cas9 off-target effects in human cells. Nat. Methods 12, 237–243. 25664545 10.1038/nmeth.3284

[bib15] Li M. , Duan L., Li Y. et al. (2019). Long noncoding RNA/circular noncoding RNA–miRNA–mRNA axes in cardiovascular diseases. Life Sci. 233, 116440. 31047893 10.1016/j.lfs.2019.04.066

[bib16] Lin F. , Chen H.W., Zhao G.A. et al. (2020). Advances in research on the circRNA–miRNA–mRNA network in coronary heart disease treated with traditional Chinese medicine. Evid. Based Complement. Alternat. Med. 2020, 8048691. 32148552 10.1155/2020/8048691 PMC7048918

[bib17] Liu C.X. , Li X., Nan F. et al. (2019). Structure and degradation of circular RNAs regulate PKR activation in innate immunity. Cell 177, 865–880. 31031002 10.1016/j.cell.2019.03.046

[bib18] Liu Y. , Mao S., Huang S. et al. (2020). REPAIRx, a specific yet highly efficient programmable A > I RNA base editor. EMBO J. 39, e104748. 33058207 10.15252/embj.2020104748 PMC7667880

[bib19] Lu T.X. , Rothenberg M.E. (2018). MicroRNA. J. Allergy Clin. Immunol. 141, 1202–1207. 29074454 10.1016/j.jaci.2017.08.034 PMC5889965

[bib20] Memczak S. , Jens M., Elefsinioti A. et al. (2013). Circular RNAs are a large class of animal RNAs with regulatory potency. Nature 495, 333–338. 23446348 10.1038/nature11928

[bib21] Panda A.C. (2018). Circular RNAs act as miRNA sponges. Adv. Exp. Med. Biol. 1087, 67–79. 30259358 10.1007/978-981-13-1426-1_6

[bib22] Quinn J.J. , Chang H.Y. (2016). Unique features of long non-coding RNA biogenesis and function. Nat. Rev. Genet. 17, 47–62. 26666209 10.1038/nrg.2015.10

[bib23] Savva Y.A. , Rieder L.E., Reenan R.A. (2012). The ADAR protein family. Genome Biol. 13, 252. 23273215 10.1186/gb-2012-13-12-252 PMC3580408

[bib24] Senturk S. , Shirole N.H., Nowak D.G. et al. (2017). Rapid and tunable method to temporally control gene editing based on conditional Cas9 stabilization. Nat. Commun. 8, 14370. 28224990 10.1038/ncomms14370 PMC5322564

[bib25] Vo J.N. , Cieslik M., Zhang Y. et al. (2018). The landscape of circular RNA in Cancer Cell 176, 869–881. 10.1016/j.cell.2018.12.021 PMC6601354 30735636

[bib26] Xiong D.D. , Dang Y.W., Lin P. et al. (2018). A circRNA–miRNA–mRNA network identification for exploring underlying pathogenesis and therapy strategy of hepatocellular carcinoma. J. Transl. Med. 16, 220. 30092792 10.1186/s12967-018-1593-5 PMC6085698

[bib27] Zuo E. , Sun Y., Wei W. et al. (2019). Cytosine base editor generates substantial off-target single-nucleotide variants in mouse embryos. Science 364, 289–292. 30819928 10.1126/science.aav9973 PMC7301308

