# Supplementary material for: Transcription-wide impact by RESCUE-induced off-target single-nucleotide variants in mammalian cells
Source: J Mol Cell Biol. 2023 Feb 23;15(2):mjad011. doi: 10.1093/jmcb/mjad011 (PMC10485882; doi:10.1093/jmcb/mjad011)
Supplement: mjad011_Supplemental_Files [file mjad011_supplemental_files.zip › Supplemental information.docx]

Supplemental information


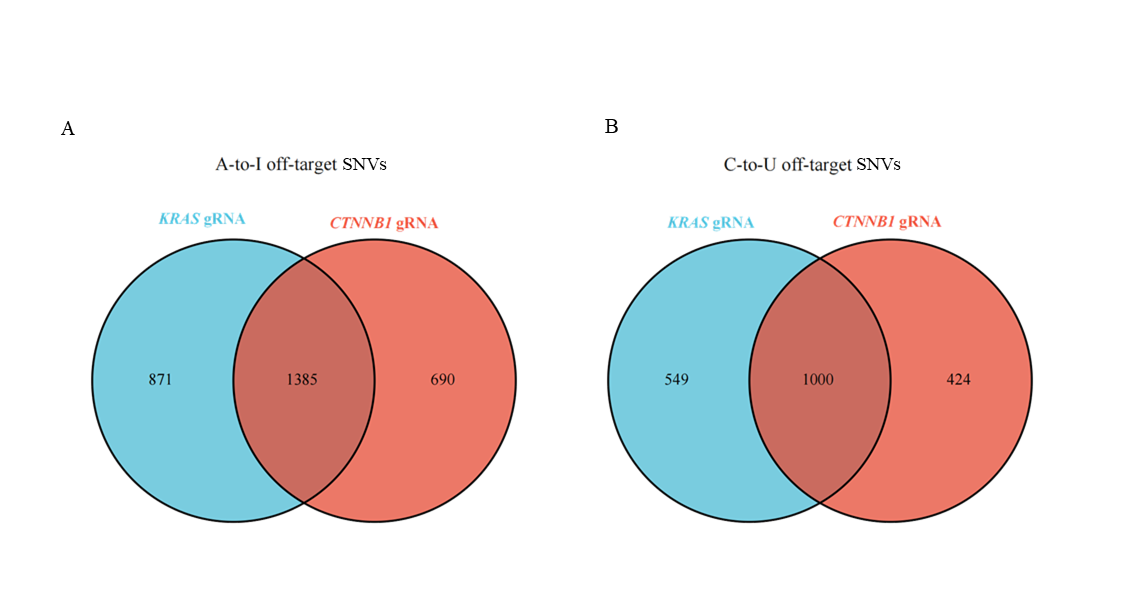


**Supplementary Figure S1** The distribution of RNA off-target SNVs between editing groups.

A. The distribution of A-to-I RNA off-target SNVs between *CTNNB1* and *KRAS* editing groups.

B. The distribution of C-to-U RNA off-target SNVs between *CTNNB1* and *KRAS* editing groups.


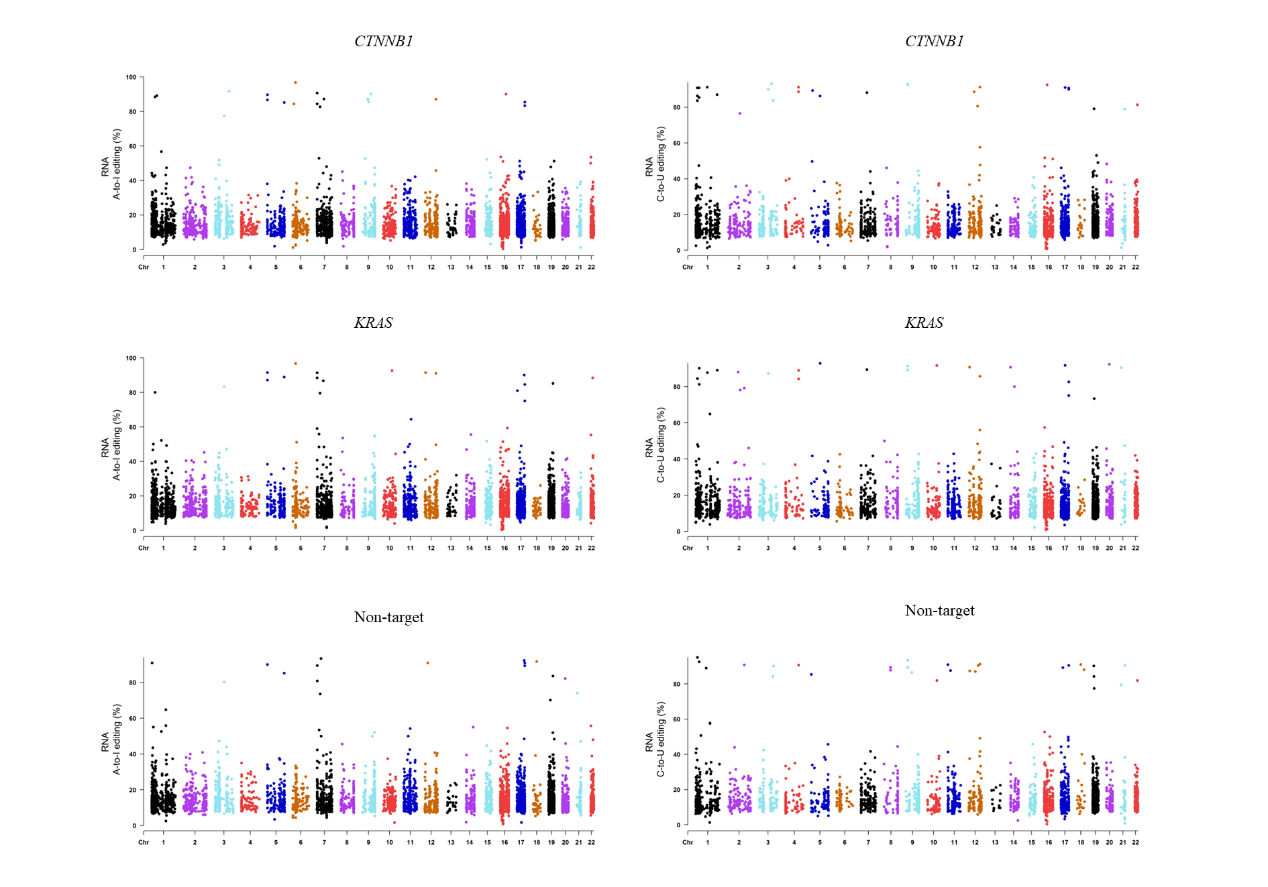


**Supplementary Figure S2** The distribution of A-to-I and C-to-U RNA off-target SNVs in chromosomes. *CTNNB1*, the *CTNNB1* RNA editing group; *KRAS*, the *KRAS* RNA editing group; non-target, the non-target RNA editing group.


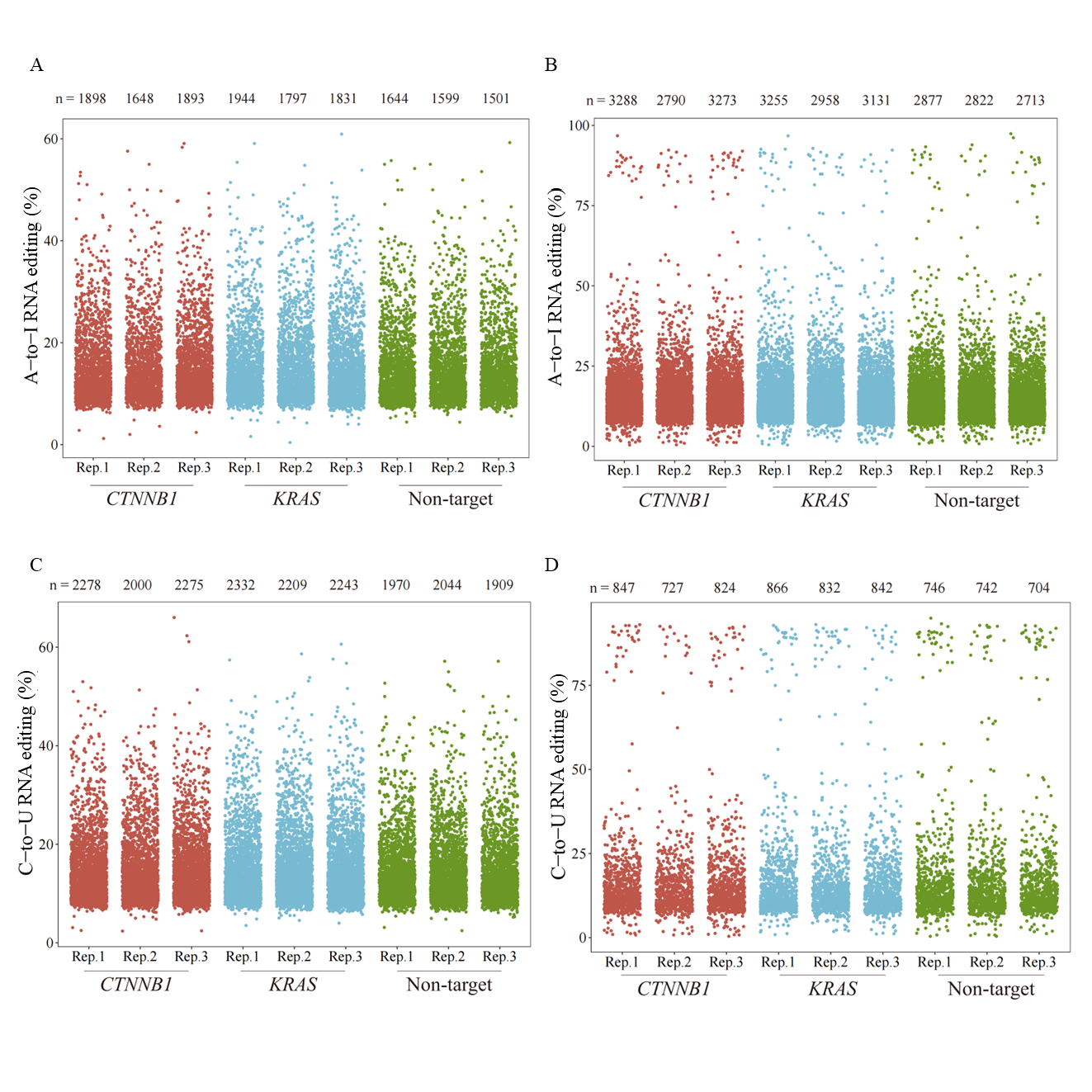


**Supplementary Figure S3** The statistics of total differentially expressed RNA associated RNA off-target SNVs.

A. The statistics of total differentially expressed RNA associated A-to-I RNA off-target SNVs appeared in *CTNNB1* and *KRAS* RNA editing groups and non-target editing group.

B. The statistics of total differentially expressed RNA unassociated A-to-I RNA off-target SNVs appeared in *CTNNB1* and *KRAS* RNA editing groups and non-target editing group.

C. The statistics of total differentially expressed RNA associated C-to-U RNA off-target SNVs appeared in *CTNNB1* and *KRAS* RNA editing groups and non-target editing group.

D. The statistics of total differentially expressed RNA unassociated C-to-U RNA off-target SNVs appeared in *CTNNB1* and *KRAS* RNA editing groups and non-target editing group.


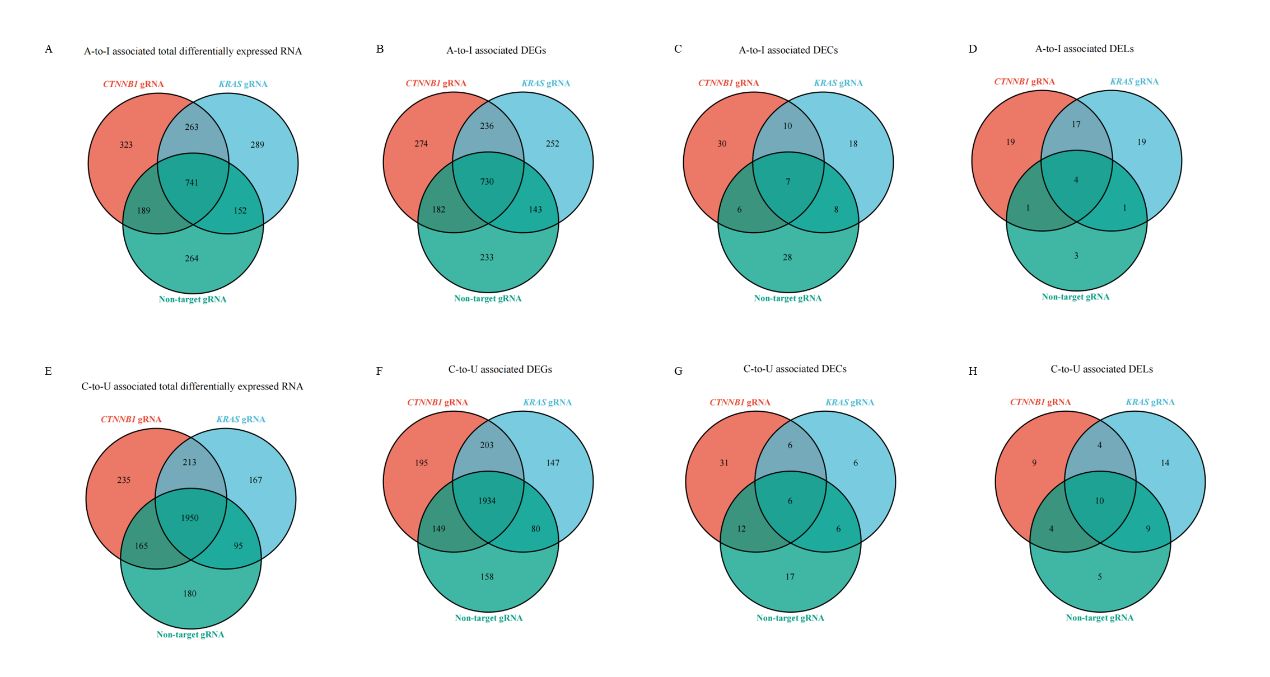


**Supplementary Figure S4** The distribution of differentially expressed RNA among RNA editing and non-target editing groups.

A. The distribution of A-to-I associated total differentially expressed RNA among *CTNNB1*, *KRAS* editing groups and non-target editing groups.

B. The distribution of A-to-I associated DEGs among *CTNNB1*, *KRAS* editing groups and non-target editing groups.

C. The distribution of A-to-I associated DECs among *CTNNB1*, *KRAS* editing groups and non-target editing groups.

D. The distribution of A-to-I associated DELs among *CTNNB1*, *KRAS* editing groups and non-target editing groups.

E. The distribution of C-to-U associated total differentially expressed RNA among *CTNNB1*, *KRAS* editing groups and non-target editing groups.

F. The distribution of C-to-U associated DEGs among *CTNNB1*, *KRAS* editing groups and non-target editing groups.

G. The distribution of C-to-U associated DECs among *CTNNB1*, *KRAS* editing groups and non-target editing groups.

H. The distribution of C-to-U associated DELs among *CTNNB1*, *KRAS* editing groups and non-target editing groups.


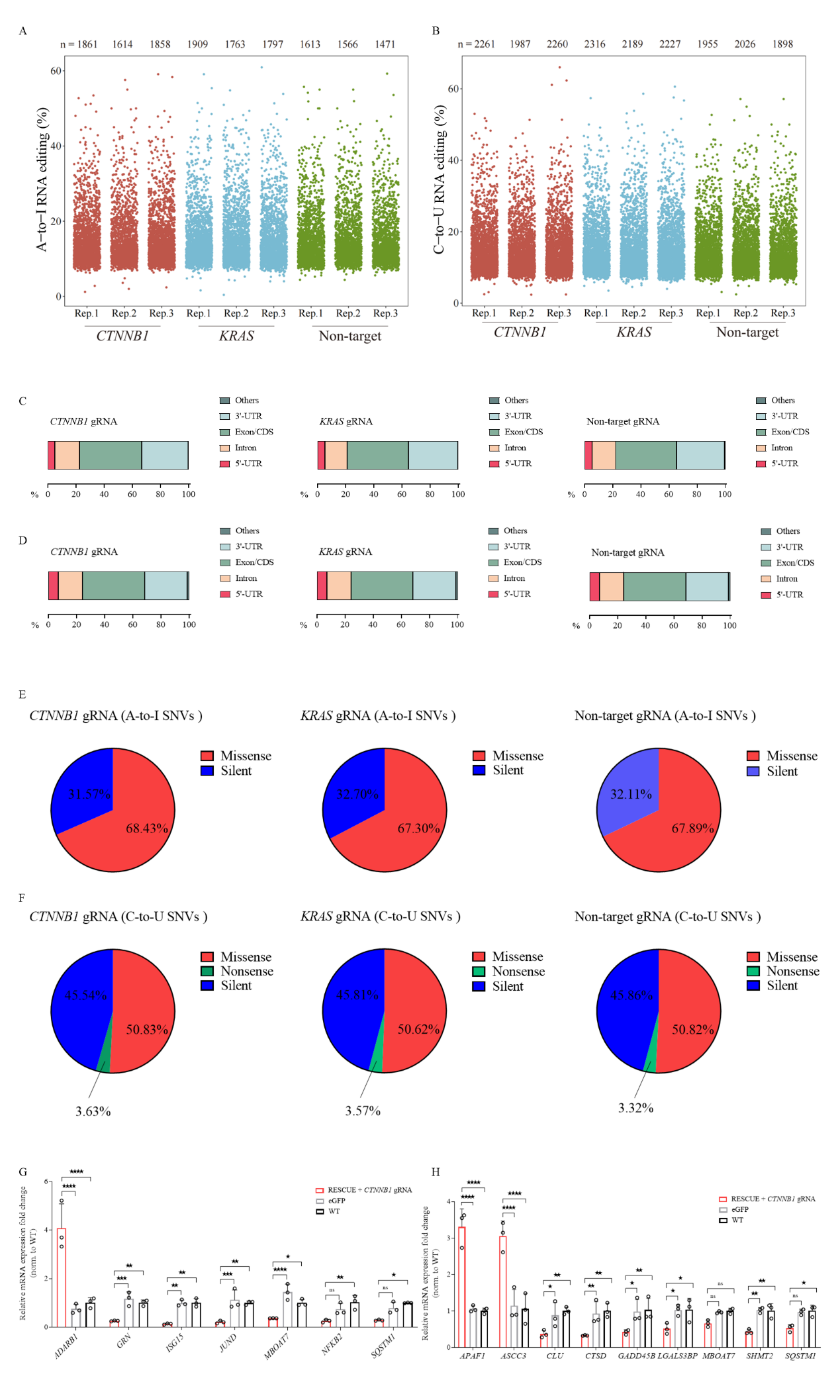


**Supplementary Figure S5** RNA off-target SNVs impact the mRNA expression.

A. The statistics of DEGs associated A-to-I RNA off-target SNVs appeared in *CTNNB1* and *KRAS* RNA editing groups and non-target editing group.

B. The statistics of DEGs associated C-to-U RNA off-target SNVs appeared in *CTNNB1* and *KRAS* RNA editing groups and non-target editing group.

C. The distributions of A-to-I RNA off-target associated DEGs in *CTNNB1*, *KRAS* and non-target editing groups. Others represent the “Up 10 kb” and “Down 10 kb” mRNA regions.

D. The distributions of C-to-U RNA off-target associated DEGs in *CTNNB1*, *KRAS* and non-target editing groups. Others represent the “Up 10 kb” and “Down 10 kb” mRNA regions.

E. The functional class of DEGs associated A-to-I RNA off-target SNVs in *CTNNB1*, *KRAS* and non-target editing groups.

F. The functional class of DEGs associated C-to-U RNA off-target SNVs in *CTNNB1*, *KRAS* and non-target editing groups.

G. Relative expression detection of the A-to-I off-target SNVs associated DEGs in *CTNNB1* editing group.

H. Relative expression detection of the C-to-U off-target SNVs associated DEGs in *CTNNB1* editing group.


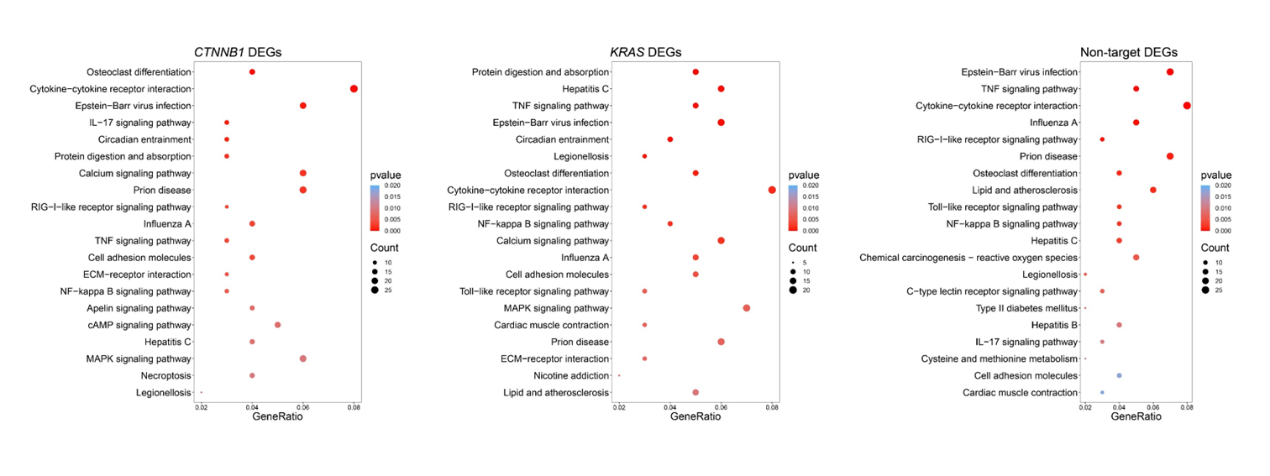


**Supplementary Figure S6** Biological function and KEGG pathway analysis of DEGs. Top 20 terms were showed.


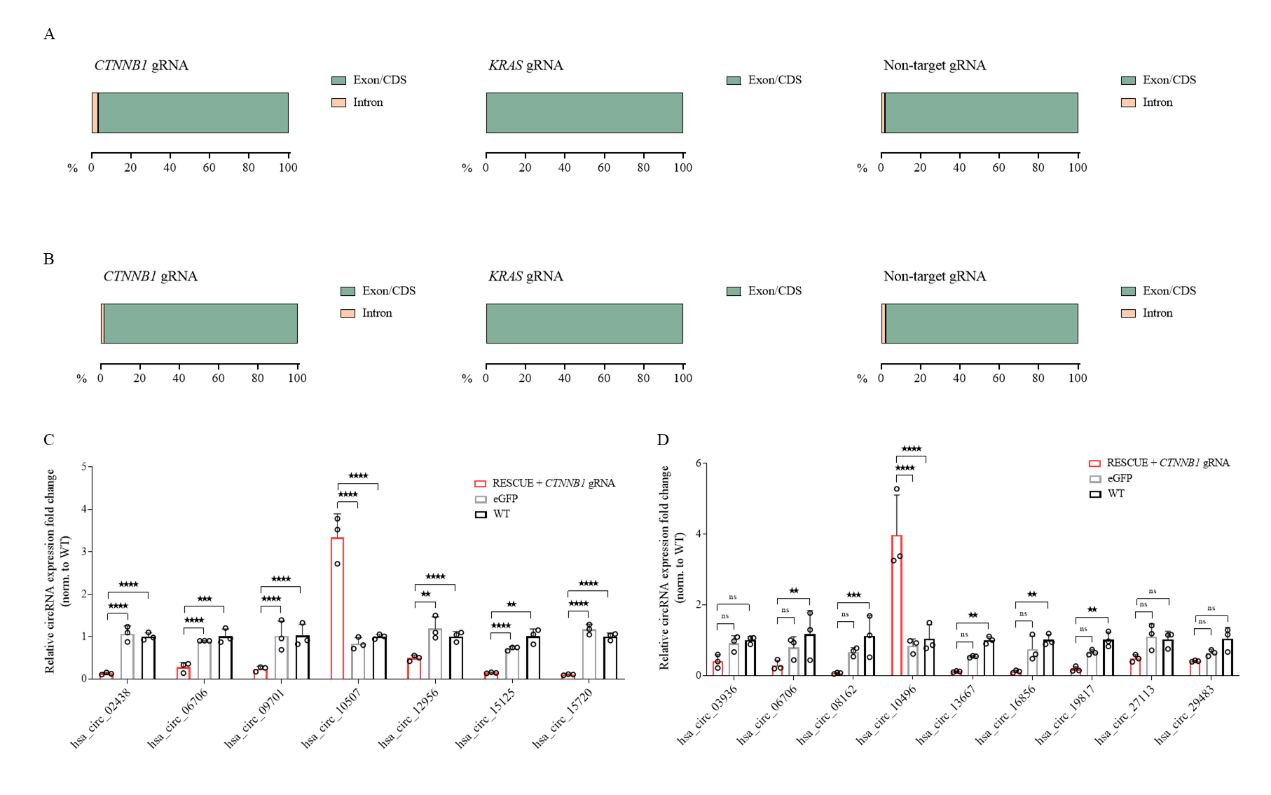


**Supplementary Figure S7** RNA off-target SNVs impact the circRNA expression.

A. The distributions of A-to-I RNA off-target SNVs associated DECs in *CTNNB1*, *KRAS* and non-target editing groups.

B. The distributions of C-to-U RNA off-target SNVs associated DECs in *CTNNB1*, *KRAS* and non-target editing groups.

C. Relative expression detection of the A-to-I off-target SNVs associated DECs in *CTNNB1* editing group.

D. Relative expression detection of the C-to-U off-target SNVs associated DECs in *CTNNB1* editing group.


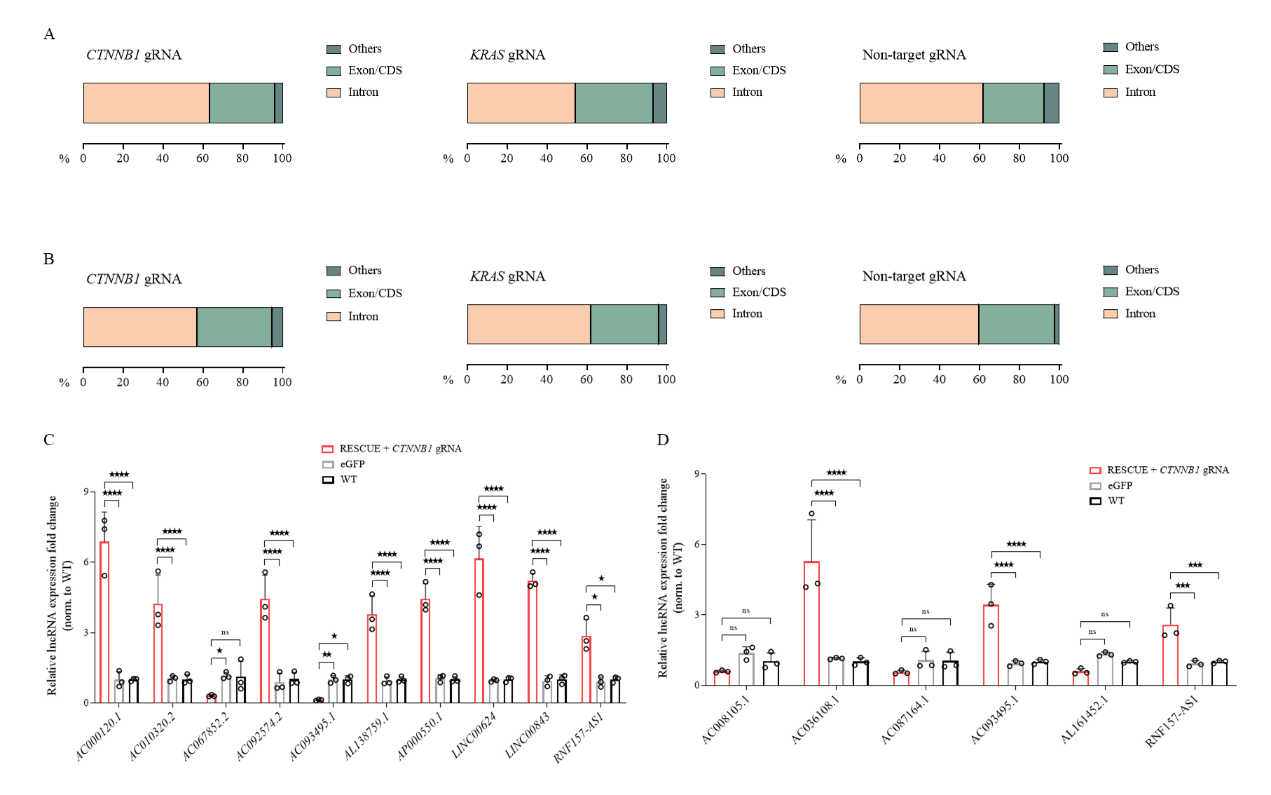


**Supplementary Figure S8** RNA off-target SNVs impact the lncRNA expression.

A. The distributions of A-to-I RNA off-target SNVs associated DELs in *CTNNB1*, *KRAS* and non-target editing groups.

B. The distributions of C-to-U RNA off-target SNVs associated DELs in *CTNNB1*, *KRAS* and non-target editing groups.

C. Relative expression detection of the A-to-I off-target SNVs associated DELs in *CTNNB1* editing group.

D. Relative expression detection of the C-to-U off-target SNVs associated DELs in *CTNNB1* editing group.


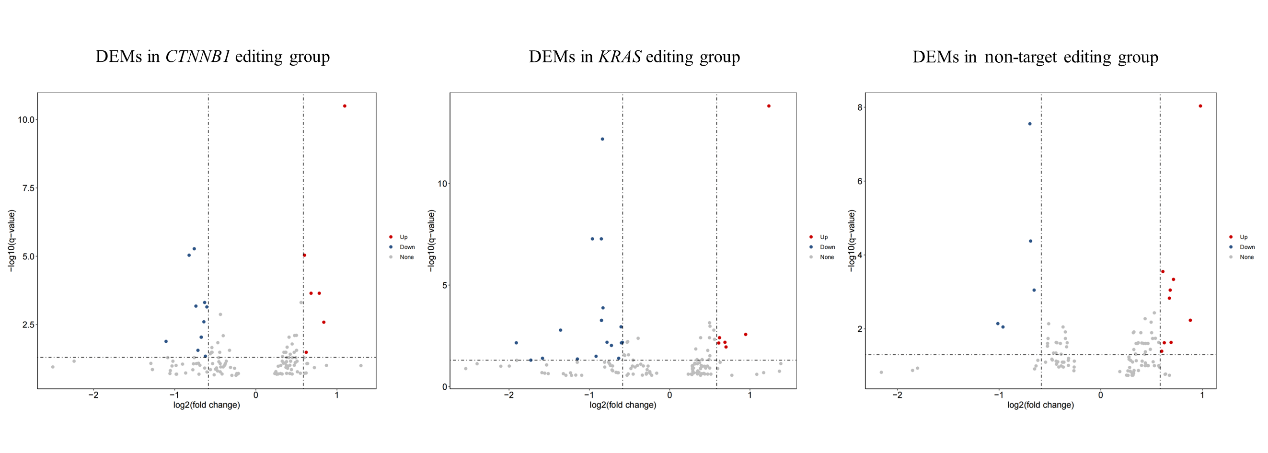


**Supplementary Figure S9** Identification of differentially expressed miRNAs in *CTNNB1*, *KRAS* and non-target editing groups. q value < 0.05, log_2_ FC > 1.5.

**Supplementary Table S1** The sequences of circRNA expression in ADAR2, dCas13b, RESCUE and eGFP expression cells detection Q-PCR primers.

| **circRNA** | **Primer F (5’-3’)** | **Primer R (5’-3’)** |
| --- | --- | --- |
| *HAUS4* | AGCAAGAAGTAGAGGAGCAGC | TGCTAGGGTGAGGCTTAAGC |
| *CCDC18* | GCTGATTCAAGAGGAGCTGC | TGCAGTCAGGTTTGCATGAAG |
| *NUP107* | TTCTTATCCTGGGAGACATTGATGG | CCAGATCATCAAGGGGCATTTTC |
| *PPP1CB* | GCTGGGGAGAAAATGATCGTG | TGCTCCATAGATTGCAGGTCTG |
| *TCF25* | TCCACTGGCGTATTTCCTGC | AGCTGCAGGAGTGAGTCAAC |
| *NEIL3* | ACAGCCCAATACTCATCACCAG | CACGGGTACTTCATTAAGTGGC |
| *AKR1A* | GAAGACTCTGGCTGACCTCC | AGCCTACGCTAAGGGCATAC |
| *DCK1* | GGATGAGAGTTACCTGCGGC | AATGTAGGTGCCAGCCTCAC |
| *BRIP1* | TCTGTGTGCCAGACTGTGAG | ACACCAAGTTCTGACGAAAAGG |
| *KIAA0368* | TGTGGCGAGTTCGAGAATCC | GTGTTTTGCCAAGAGCTCCC |
| *GAPDH* | CCACTCCTCCACCTTTGAC | ACCCTGTTGCTGTAGCC |

**Supplementary Table S2** Guide sequences used for endogenous gene editing.

| **Targeted gene** | **Motif** | **Base flip** | **Spacer sequences** |
| --- | --- | --- | --- |
| *CTNNB1* | UAC | A/11 | TGGTGCCACTACCACAGCTCCTTCTCTGAG |
| *CTNNB1* | UAC | A/11 | TGGTGCCACTACCACAGCTCCTTCTCTGAG |
| *CTNNB1* | UAC | A/11 | TGGTGCCACTACCACAGCTCCTTCTCTGAG |
| *KRAS* | UAU | A/25 | TTCAGAATCATTTTGTGGACGAATATGATC |
| *KRAS* | UAU | A/25 | TTCAGAATCATTTTGTGGACGAATATGATC |
| *KRAS* | UAU | A/25 | TTCAGAATCATTTTGTGGACGAATATGATC |

**Supplementary Table S3** The Q-PCR primer sequences of verified differential expressed mRNA, circRNA and lncRNA.

| **Gene Name** | **Primer F (5’-3’)** | **Primer R (5’-3’)** |
| --- | --- | --- |
| *ADARB1* | GGATATAGAAGATGAAGAAA | ATTGGAGAGCTGAGAGCCCT |
| *ACOT12* | CTGCTCAAGTGGATCGACAC | CTAGCTGTCTCCTCAAACTG |
| *GCGR* | TGCTGCTGTTGCTGCTGCTG | CAGCAGGCTCAGGTTGTGGT |
| *CLEC2A* | CAGAGCTGATGGCTTCATAC | TTGGACCATGTGGCTATCAT |
| *SCRG1* | GATCTGTTACTGCAACTTCA | TCATTGATTGTTGCAAGGAA |
| *CTCFL* | TACAAGCTGAAACGCCACAT | TATTTTCATGGTCCCGCTCT |
| *FGF6* | AAAGGAAGATTGTACGCAAC | TTGGTACAAGTCTGACTCGT |
| *PLA2G5* | ATGAAAGGCCTCCTCCCACT | CCCTGTCACCTTCTCGATCA |
| *TMEM215* | AGCCTGGCTTCTTTCCCTAC | GTTTCACTCCATCCAGGTTG |
| *CELA2A* | ATGATAAGGACGCTGCTGCT | CTCGCTTCTTCACCGCCAAC |
| *CSAG3* | AAGGCATCCTCTCAACCCTG | TGGAACTTCCTTGATGGGTC |
| *CCL5* | ATCCTCATTGCTACTGCCCT | CGGGCAATGTAGGCAAAGCA |
| *IFNB1* | CATGAGCTACAACTTGCTTG | GTCCTTGAGGCAGTATTCAA |
| *IL32* | GTGATGTCGAGCCTGGCAGA | TCAAGTAGAGGAGTGAGCTC |
| *B3GALT5* | CTTGGTCTTGGACTGAGCCA | CTGTCAGTGTAGAATGCTCC |
| *IFIT2* | GAGGAAGATTTCTGAAGAGT | GTAGGCTGCTCTCCAAGGAA |
| *KRT75* | GCTGCTGAGAATGAATTTGT | TCTCCTCGGGCAGAGATTTG |
| *IFIT3* | AAACAAAATCAACCGGGACC | CTGTGGAAGGATTTTCTCCA |
| *CXCL10* | TCTGATTTGCTGCCTTATCT | TTACTAATGCTGATGCAGGT |
| *OASL* | GGGTGCTGAAGGTAGTCAAG | GCTGTGGAAACAGCTCAGAA |
| *GRN* | TCCTGCTTCCAAAGATCAGG | TCGAACTGACTATCAGGGCA |
| *SQSTM1* | AAGGTGAAACACGGACACTT | ATGGACCAGAAGCTGATTCT |
| *MBOAT7* | GAGCAGCTCAGACCATGTCG | CAGGACCGGCTTTCTTAAAG |
| *JUND* | AACAAACGTTGGTTGTGTGT | GGAGCGAGATCGAGGAAAGG |
| *ISG15* | CACCTACGAGGTACGGCTGA | AGAACAGGTCGTCCTGCACA |
| *CLU* | CAGACAATGAGCTCCAGGAA | CTGTTTCACCCCGTTGACAG |
| *LGALS3BP* | TGCTGGTTGCAGGAACCCAA | TGTAGAAGATCTCCACGCGG |
| *SHMT2* | TACAACCAGCTGGCACTGAC | AGTCAATGAGGCGAGCATAG |
| *GADD45B* | GCTCTAGCTCTGTGGGAAGG | ATAATCCACAGTGGATGCGG |
| *APAF1* | GTCACCATACATGGAATGGC | CCCTGGGAAACAACCTTCTA |
| *ASCC3* | AGTTGGCATGGCTTTACCTC | CCATGTCAGGCCCAAATCTA |
| hsa_circ_14850 | GAAGAGGCAAAGTCTGTGGC | CTGCACTTCTGCTCCATTTC |
| hsa_circ_17142 | GGACTGAAGATTGGTGCAAT | TCTGGCCAAGCTCTCGAATC |
| hsa_circ_07102 | CACATATAACTGCAGCAGCC | AGCATTCCTGAGATCCTTTC |
| hsa_circ_03171 | ATATGGAGGAGGACGTGTAT | TTTGCGCAGCCGGTACTGCA |
| hsa_circ_03846 | ACACTTCCTCCTGGATGATG | CTTCTGCTGCTGAGGTAAGT |
| hsa_circ_30495 | ACTGGGAGGCTACTTACAGT | CTGGACTTCTACTGGATGAT |
| hsa_circ_08238 | GTGCTGCAATCATCCGTACC | ATCATCTGCCTCCTCATCAG |
| hsa_circ_21135 | TGGCAACTTCTGGTAATGGT | TTCTCCATCTTCAGTGCCTC |
| hsa_circ_20660 | GAGCCGGCAAAACAGAAAAT | AGTGAACAGTTATCTTCCAC |
| hsa_circ_00802 | CATCCGAAAGGAGCCAAAAC | TGTTGCTACAATCAGCTCCG |
| hsa_circ_17979 | ACTTCAACGACCCCAAGTAC | GCATCCAAGTGTGGCACTTT |
| hsa_circ_05313 | CGGCTCCTCAGAAGATTCCT | TGTTTCTCTGTCCAGGCCGA |
| hsa_circ_01486 | GAACGTTTGTCACACTTCCG | GGACTCCAGCTGCTTGAAGT |
| hsa_circ_25324 | GCGTCCTGGAAAACTTCACC | GTACCCTGGGAGGTCAGTAG |
| hsa_circ_31088 | CAGTACGTCAGCTGCCATGC | TCAGCTTTCCAGATGCAGAG |
| hsa_circ_21006 | CAGCAAGCACATAGTGGAGC | AGGATAGGGTGGTTCGGGAA |
| hsa_circ_09334 | GTACCCAAAAGGTCACCCAC | ACAGTACAATTCGGGGCCAC |
| hsa_circ_06867 | GATATGATCGTGGGTATGACAG | GTGTACAAACTGAGGCCAAA |
| hsa_circ_05882 | CCTAAGCAAATTGAAGAACG | CCCACAGTTCTCTGACTTCT |
| hsa_circ_00856 | GCGGAGGTGTCTGAACTAAA | ACGAGCTACCTTTTCAGATG |
| hsa_circ_06706 | GCTGCCTTGTACCCACATCT | GGAAGATGTCATCCTTCACG |
| hsa_circ_09701 | ATGAATCTCCCAGTCCCTGA | AATTTGCCACCAAGTCTTCT |
| hsa_circ_02438 | CGGAGGATCCGAGTGTGAAT | GCCATCGCAGATCACATTGG |
| hsa_circ_17700 | TGCATCTGAAATGATTGCTG | CCTGAATGTTCACTTACACC |
| hsa_circ_15125 | TTCAACTTCACCTCGGCAAC | CTGGAGGGAAGGAATCATCA |
| hsa_circ_12956 | CCTCACTTAGGTCTTGGGTC | TGGCAGGCTCTTGGTTCCTA |
| hsa_circ_15720 | CTTAGCCCTCTGGATGAGGA | GGAAGATGTCATCCTTCACG |
| hsa_circ_10507 | ATGAGGCCATCTATCCCGAG | AGAGAAGGCTCTTCTTTGGG |
| hsa_circ_19817 | CAGTCAGCCTCAGAGGTTGA | GCATTTTGTAGAGGGAGTGC |
| hsa_circ_29483 | GCTGCTGGAAATGCTGACTT | GATAAGAGTGCTGCAGAGCT |
| hsa_circ_03936 | TCTGTCACCATCACAGGCAG | GACGCAGCATCTTCCTGTGA |
| hsa_circ_10496 | CCTCCTGCAAGATGATGTTG | AAGGCCTACTTGTGTGACAC |
| hsa_circ_16856 | AGTCGGTGGTACCTGAGATG | GCAGCCCAATCAGGTCAAAG |
| hsa_circ_08162 | CCTTGTCACTGGTCTGAGGT | TCACAAAGGCACTCCAGGTG |
| hsa_circ_13667 | CCTGGGTTTGACTGGTTCTT | CACTGAGTCACCCCAACTTT |
| hsa_circ_27113 | ACCCTATGGAGGTCCTGGAC | CATGCCCGTCAGCTTCTCTT |
| *ELFN1-AS1* | GCCTCAGCCACAATCGTAAT | ACTTGGAGCAGCCACTTAGA |
| *AL583810.1* | TAGCCCTGCCAGAGAGTAGA | GATCACACAGACCTGCTCTG |
| *AC074389.1* | TTAAGGTGATGGCATTCCCC | CCACAGACTCAGCAGGGAGC |
| *GLIS2-AS1* | TAGGTTCCTGCTCTCCTGAA | GCCCCATCTTACAGAGGACC |
| *GOLGA8M* | ACTTCGCTTGTCCTCTCTGC | CTTTGCACGATGACTGGTTC |
| *AC002064.1* | ACATGATGCCTACCCGATCC | CGTGCCACTTCATGTATGTC |
| *AL606491.1* | AGGCAGTAGCTGACCTGGAC | CTCTTCCTGAGTCCTGGGCA |
| *AC099795.1* | CTCACCTGAACTGTGAGAAG | TGGAAAGCTGGACTGGAGTT |
| *AL136115.2* | TAGAAAGGAGAGGATGAGGT | ATCTCGGCTCACTGCAAGCT |
| *LINC00311* | CAGGCTATCGTCCTGCAGCT | CGCCGTTCTTCATCACACTT |
| *LINC00944* | GACTAGACGCACATCAGGAA | TATCATTCTCTCTCTCTGTC |
| *LINC02243* | AGACCTTCCTCTCTCTGGCT | TCACGCAGGAAGAATTCTCT |
| *EGOT* | AGATGGAAGACTCCTAGGAT | CCATCTTTAAGGAGGAGGCC |
| *LINC00943* | TGTGTGTGAGTGTACATGTC | GTGTGCACTTGGAGAGAGTT |
| *LINC00908* | TGAGGCCTAGTGCTCCTAGC | CTAGGATGTCCAGGCTCTGC |
| *LINC02137* | AAGTGATTCTCCTGCCTCAG | CCTCTCTGAGACGTCATGTC |
| *AL132655.2* | TGTCATGTCAGCTGCTGAGT | CTGAGGGTGGGAGGTAGGTA |
| *MIR503HG* | ATCCGGCTTCCTCCAGTGGA | GTACTGCTAACTGGAGATGC |
| *ERICD* | TCTGCATATGGGTGCAAGGC | AACTAGTGACCACCCTGCTG |
| *AL023284.4* | AATAGTCACCGTGAGTCAGG | GGCTTCAGAGATGCAGCTGA |
| *AC067852.2* | CAGCCTGGGCAACATAGTGA | CTCCAGGAGCTGGGACTACA |
| *AC093495.1* | GTGTTGTCTTTCCAACAGAG | TGTTTTCTCCCGTATGGGTT |
| *AL138759.1* | CAAGATTGCATCACCGCACT | CAGTGACTCAGTATTACCAG |
| *AC000120.1* | CAGGTGCTCTGAGTTTAACC | TCATGCACTAAACTGGAAGC |
| *LINC00624* | GGCTTCATTCCTGGAGTGAG | TCCGGAGTTTGTTCCTTCAG |
| *RNF157-AS1* | GGAGGTCCTCTGATCCTTGA | GACTGACATCGGAGAAGTCT |
| *AP000550.1* | TACAGGTGTGTGCCACCACA | GGAGTTCGAGAGCAGCTTGG |
| *AC010320.2* | GAGCCAAGATCACACCACTG | TGCCCAGCCTCAGATTCTTC |
| *AC092574.2* | TCAGCCTTCCGAGTAGCTGG | GATTGAAAACATCCTGGCCC |
| *LINC00843* | CGTAAGTATGGGGCCTAGCT | AACATAAAACATGGGACGCG |
| *AC093495.1* | GTGTTGTCTTTCCAACAGAG | TGTTTTCTCCCGTATGGGTT |
| *AC008105.1* | TTCCTCAGCCTCCTGAGTAG | CCATCCTCGCTAACACGGTA |
| *AC036108.1* | GCATGCCACCATGTCTGATT | TTGAGATGGGCAGATCACCT |
| *AC087164.1* | ATTAACTGGCCATGGTAGCA | AACTCCTGGGCTCAAGTGAT |
| *AL161452.1* | GAAACAGATGGAAGGTGAGC | AGTGACCTGAGTCCTGCAGA |
